# Palliative care made visible: Developing a rural model for the Western Cape Province, South Africa

**DOI:** 10.4102/phcfm.v11i1.2147

**Published:** 2019-10-31

**Authors:** Victoria O’Brien, Louis S. Jenkins, Margie Munnings, Hilary Grey, Zilla North, Helise Schumann, Elmari De Klerk-Green

**Affiliations:** 1 Improving Global Health, National Health Services, Thames Valley and Wessex Leadership Academy, Winchester, United Kingdom; 2 Department of Family and Emergency Medicine, Western Cape Department of Health, George Regional Hospital, Garden Route District, George, South Africa; 3 Department of Family and Emergency Medicine, Faculty of Health Sciences, Stellenbosch University, South Africa; 4 Division of Primary Health Care, University of Cape Town, Cape Town, South Africa; 5 Western Cape Department of Health, George Regional Hospital, Garden Route District, George, South Africa; 6 Department of Palliative Care, Knysna Sedgefield Hospice, Knysna, South Africa; 7 Western Cape Department of Health, Garden Route and Central-Karoo Districts, George, South Africa

**Keywords:** family medicine, rural health, palliative care, integrated care, multi-professional

## Abstract

**Introduction:**

Caring for people with life-threatening illnesses is a key part of working in health care. While South Africa launched the National Policy Framework and Strategy for Palliative Care 2017–2022, integrating palliative care into existing public health care is in its infancy. Most patients in the Western Cape have poor access to palliative care, an inequality felt hardest by those living in rural areas.

**Building the model:**

In 2018, with district wide institutional managerial support, a palliative care model for rural areas was initiated in the Western Cape. The process involved setting up hospital- and community-based multi-professional palliative care teams, initiating weekly palliative care ward rounds, training champions in palliative care and raising awareness of palliative care and its principles.

**Discussion:**

Establishing regular ward rounds has changed the way patients needing palliative care are managed, particularly in challenging the mindsets of specialist departments. The emergence of the multi-professional team listening and planning together at the patient’s bedside has restored some of the dignity and ethos of patient-centred care, which is a core principle of the provincial Health Care 2030 vision.

**Conclusion:**

In a short time period, we have managed to build a service that aims to improve care for palliative patients in rural areas. Its strength lies in a multi-professional patient-centred approach and improved communication between different components of the health system, providing a more seamless service that supports patients when they need it most.

## Introduction

The World Health Organization (WHO) estimates that each year 40 million people worldwide need palliative care, with only 14% receiving it.^1^ Of these, 78% live in low- or middle-income countries.^1^ An increase in the prevalence of chronic diseases is causing patients to live longer, with life-limiting incurable illnesses.^2^ A recent study in Malawi described the impact of end-stage renal disease on patients and families^3^; however, there remains a paucity of publications on palliative care in Africa. While Kenya, Uganda and South Africa have often led the way,^4^ the need for an integrated model of care within government-run health care systems has prompted the authors to describe a South African model.

Palliative care is:

[*A*]n approach that improves the quality of life of patients and their families facing the problems associated with life-threatening illness, through the prevention and relief of suffering by means of early identification and impeccable assessment and treatment of pain and other problems, physical, psychosocial and spiritual.^5^ (p. 1)

The 2014 resolution of the World Health Assembly (WHA) acknowledged that:

[*P*]alliative care is an ethical responsibility of health systems, and it is the ethical duty of health care professionals to alleviate pain and suffering, whether physical, psychosocial or spiritual, irrespective of whether the disease or condition can be cured.^6^ (p. 1)

In 2015, South Africa ranked 34th in the Economists ‘Quality of Death Index’, the highest-ranking African country, a reflection that pockets of excellence exist.^7^ Palliative care services started in the 1980s when non-governmental organisations (NGOs) began caring for patients with AIDS and cancer. The National Hospice and Palliative Care Association (HPCA) started in 1986 supports more than 150 000 people with life-limiting conditions annually. It reaches around 40% of those in need of palliative care.^7^

Apart from AIDS and tuberculosis, South Africa has a growing number of people living with chronic non-communicable diseases, causing increasing numbers of people to live with life-limiting illnesses.^8^ In 2016, 26.2% of deaths were attributable to cardiovascular disease, cancer, diabetes or chronic respiratory diseases.^2^

South Africa launched the National Policy Framework and Strategy for Palliative Care (NPFSPC) 2017–2022 in response to the WHA’s Resolution 67.19 (of which South Africa is a co-sponsor).^9^ It states that palliative care services are crucial in managing the pain and suffering associated with life-threatening illness. The framework prioritises palliative care and training of health workers involved in palliative care. It emphasises that a gold-standard service ‘will address issues of universal health coverage and the need to reduce suffering and promote development and dignity for all’, as a human right.^9^

The Western Cape Province has several metropolitan hospital-based palliative care services.^10^ In the Garden Route district, Knysna Sedgefield Hospice provides an exceptional service to patients.^11^ Apart from these, very little other comprehensive end-of-life care exists in the province. Support from NGOs, for example, the Cancer Association of South Africa (CANSA), is invaluable to those reached, but most patients in the Western Cape have poor access to palliative care. Isolated pockets of excellence fail to reach some of the most vulnerable and isolated patients, highlighting the need for an integrated system that can ensure more equitable care.

This article describes the innovative development and implementation of a palliative care model in a rural district of South Africa. The model focuses on successes and lessons learnt from creating multi-professional palliative care teams at a regional and district hospital, with the aim to showcase a replicable solution to improve palliative care for all patients.

## Early beginnings

The project initially focussed on George hospital, a 272-bed regional referral hospital. Eight clinical departments care for 160 000 outpatients annually reach out to 10 district hospitals and train Cape Town and Stellenbosch University students in the health sciences. It services the George sub-district and is the referral centre for the Garden Route and Central Karoo districts, with a combined population of 685 000 people.^12^

In 2018, the hospital management supported the appointment of a sessional palliative care specialist. The provincial chief director of rural health and the local district manager requested a situational analysis to determine existing services and role players, and in August 2018, the first stakeholder meeting was convened.

Through an existing 5-year collaboration with the ‘Improving Global Health (IGH) through Leadership Development’ programme in the United Kingdom, as part of a collaborative leadership development initiative, young health professionals (fellows) have rotated 6-monthly, addressing locally identified quality improvement projects. A fellow was allocated to the Palliative Care Initiative and worked with the head of family medicine and the sessional palliative care specialist to develop a rural palliative care model. Several steps were followed, including:

1. setting up a hospital multi-professional palliative care team and initiating weekly palliative care ward rounds
2. creating resources and monitoring and evaluation
3. setting up a community multi-professional team with improved communication between the hospital and the sub-district
4. training champions in palliative care
5. awareness of palliative care and its principles.

## Building the model

### The multi-professional team and palliative care ward rounds

A multi-professional team was created consisting of a palliative care trained family medicine doctor, medical officer, physiotherapist, psychologist and social worker. The ward doctor and nurse looking after the patient are encouraged to attend with the team. Together they do a weekly ward round of identified patients, using the internationally recognised SPICT^®^ tool (see Appendix A).^13^ The focused ward rounds are patient-centred, allowing the patients to express their thoughts, fears and expectations. They speak face to face with the patient at eye level at the bedside (together with the family, if possible) exploring bio-psycho-social-spiritual concerns.

### Creating resources and monitoring andevaluation

A Palliative Care Plan was developed as a standardised tool for the hospital and sub-district (see Appendix B). It is shared with the patient and regularly updated. Symptom management is optimised and follow-up arranged. All professionals use it to provide a seamless approach. A booklet of local community resources involved in the care of people at end of life was developed for staff and patients to refer to. This is also available electronically.

For monitoring and evaluation of the ward rounds, we used Epicollect5^®^, a free, mobile web-based application. The team utilised this application to collect data in real time during ward rounds on efficacy and efficiency. Preliminary data for 16 ward rounds showed that the average duration has been 98 min per round, with 62 patients consulted. The most common conditions seen were cancer (29%), cerebrovascular incidents (14%), AIDS (11%), renal failure (11%), tuberculosis (7%) and chronic obstructive pulmonary disease (7%). The average age of referred adult patients was 52 years, with 31% of patients being under 35 years old. One-third of these patients had complications from AIDS.

### Setting up a community multi-professional team, with improved communication between the hospital and the sub-district

Upon discharge, patients leave the hospital with their palliative care plan. Referrals reach the community via a generic email address which is checked by the community multi-professional team. This team is based at the sub-district hospital. While this is a tuberculosis hospital, functionally it also serves as the district hospital, in terms of its staff component and connectivity with the home-based care services. In other sub-districts, this role is fulfilled by the district hospital. The multi-professional team meets weekly, with representatives from each of the Community Day Centres, the social worker and palliative care trained physiotherapist, doctor and nurse. Referrals are made to the team from the hospital, clinics and community health workers, who visit homes in the surrounding areas. The team keeps a database of palliative patients in the area, ensuring patients are not lost in the system and building a profile of local palliative care needs.

### Training of champions in palliative care

A range of professionals from the regional hospital, sub-district clinics, health centres and the local NGOs underwent training over 5 weeks during 2018. Initial findings showed that participants reported a greater than 30% increase in how equipped they felt to use ethical principles to make a palliative care assessment, 71% felt they would volunteer to lead a palliative care team following the course and 82% felt prepared to break bad news, a 25% increase on pre-course figures. All the attendees reported enjoying networking, sharing challenging cases in a safe space and building a team with a variety of professional colleagues.

### Awareness of palliative care and its principles

Awareness of palliative care has been created at various levels. The ward rounds have provided an opportunity to share knowledge and teach colleagues palliative care through bedside role modelling. Feedback included:

‘I’ve been exposed to a more holistic approach to care and thinking about patients beyond their hospital stay.’ (Intern)

‘[*A*]ll patients could benefit from a palliative care decision making approach.’ (Intern)

‘I enjoy and learn from being part of a multi-disciplinary team’ and ‘I can approach difficult topics with confidence.’ (Team member)

Bringing together professionals for the ward rounds has increased their skills as they have learnt from each other. The training and local presentations have opened the eyes of many colleagues to the discipline and its principles, leading to the message being passed on in various work environments. This has been seen in many outcomes including better pain control for patients, with ward staff reporting an improvement in pain management from 41% to 67% since the inception of the ward rounds. Monthly sub-district palliative care meetings with a wide range of stakeholders across many disciplines have given the project structure and increased awareness. Sub-district and rural provincial meetings have created buy-in and awareness.

## A model for service provision

**FIGURE 1:**
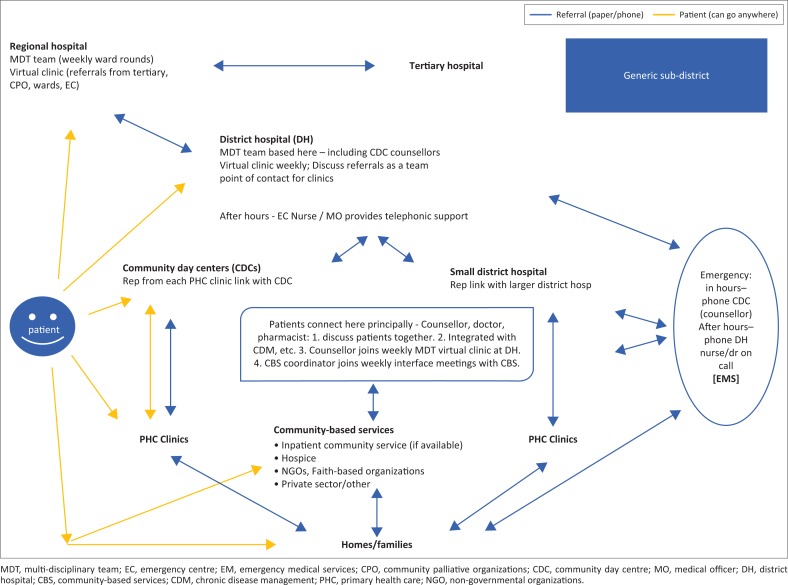
Rural palliative care model.

As a pilot for a rural palliative care model, we have attempted to build a service that could be replicated in other rural districts. Figure 1 illustrates how patients are referred as well as how they move between the various facilities in a sub-district.

The model highlights how continuity of care and communication are optimised between the regional hospital, district hospitals, clinics, NGOs, emergency medical services (EMS) and the patient’s home. The model has been shared with the rural districts and the chief director for rural health in the province. It has received widespread support, acknowledging the need for high-quality palliative care, but also seeing an opportunity to improve existing aspects of continuity of care in the management of chronic diseases across the whole health spectrum.

## Discussion

Establishing regular multi-professional palliative care ward rounds has changed the way patients needing palliative care are managed, particularly in challenging the mindsets of specialist departments. Death is often viewed as a failure, and proactive planning with patients about their wishes in their final days was not routinely addressed. Doctors in general avoided having difficult discussions about dying with patients and families, instead focusing on the medical and technical aspects of diseases. The emergence of the multi-professional team listening and planning together at the patient’s bedside has restored some of the dignity and ethos of patient-centred care, which is a core principle of the provincial Health Care 2030 vision.^14^

The illness experiences of patients (in contrast with just the disease) are much more appreciated, allowing patient autonomy, fostering respect and caring with competence and empathy.^15^ As the hospital is a training site for undergraduate health professionals, students are being exposed to the way empathy is made visible, which has become a key graduate attribute in medical schools.^16^

Initiating and sustaining the model involved a change management process, realising that changes that are accepted into people’s minds and the institutional culture are usually emergent, in response to challenges in care being delivered at the frontline.^17^ This has been the experience of this project. Patients needing palliative care were already in the system, but without clear care plans. Responding to this need, staff members were enthusiastic to improve their care and understand palliative care more. The core team all have palliative care training, and therefore, the rounds have been an opportunity to share knowledge and experience with doctors and nurses with less experience. It has been challenging to get ward teams to give up time to join the rounds as it completes with other commitments and priorities, but they have seen how shared care can strengthen their work with patients and help to build links with the community prior to discharge.

While recently described models in Cape Town focussed on establishing palliative care outpatient clinics, the rural model attempts to utilise existing structures. Ward rounds and a virtual clinic aim to minimise additional workload on already overworked health professionals and optimise feasibility.^10^

An essential element of the model’s success was organisational readiness for change at managerial level, with visionary leadership.^18^ Following on from this, a process of collaborative leadership began between key people. Passion within the hospital and sub-district emerged iteratively as the model took shape, giving momentum. We were comfortable with uncertainty, being patient and building relationships, while creating an environment of trust and mutual respect, appreciating the contributions of all the team members.^19^

The model has developed participating staff’s confidence, with not just doctors taking the lead in consultations, but an appreciation for the value of the whole teams’ skills. The psychologist, traditionally aligned to the psychiatric team, has become an integral part of the wider care of patients. The social worker has embraced technology and taken the lead in data collection. The physiotherapist reminds staff of the weekly rounds and provides a practical approach to problems. With awareness and enthusiasm growing, the Paediatrics Department has recently started referring patients. As more specialist departments are asking for consultations, the weekly morning ward rounds shifted to the afternoons, to allow departmental doctors and nurses to join, as well as overlap with families during visiting hours. These small actions are building sustainability and keeping the momentum for positive change.^17^

## Challenges and next steps

Home visits are not happening as regularly as they should. Ideally, all patients diagnosed with an end-of-life illness should receive a home visit soon after diagnosis. One can truly begin to understand the patient’s illness only by seeing the home situation of patients. By acknowledging the patient’s experience in their home and community, we can move beyond simply treating the disease. For this, a radical plan of resourcing more home-based carers and community health workers is needed.^20^ Moving forward, we hope that all palliative patients will have access to a holistic, high-quality assessment, including adequate pain control and symptom management. Patients and their families need access to a contact number for any concerns which would address anxiety and enable signposting to relevant points of contact in the health care system. The palliative team is communicating more and more via a WhatsApp^®^ group, building and maintaining awareness of palliative care needs in the community. A second cohort of health professionals from across the district has also now been trained, doubling the number of people trained in basic palliative care. Training of counsellors at the primary health care clinics, who liaise with the home-based carers, has started. Utilising available staff’s skills in the absence of enough home-based care has bridged some gaps. It has shown the value of training in upskilling professionals who have valuable soft skills. We are also collecting data on community need to help drive future service provision. Other rural districts are starting to address the needs of palliative care patients in similar ways. For sustainability and feasibility in the long run, it is important to integrate palliative care into primary health care and not have another vertical programme. The principles and logistics around palliative care are not unlike those needed, for example, chronic disease management, with patient-centred care, whole person care, whole of society approach and continuity of care accepted as the goal.

## Conclusion

In a short period of time, we managed to build a palliative care service that aims to improve care for patients in rural areas. Its strength lies in a multi-professional patient-centred approach and improved communication between different components of the health system. It provides a more seamless service that supports patients when they need it most. While more time is needed to collect data on prevalence and patient feedback, we have shown the benefits of training and collaboration between team members and ward staff. It has initiated a discussion on palliative care, challenging misconceptions and asking questions, which are ultimately benefitting patients.
